# Hypercoagulation from Concomitant Administration of Tamoxifen and Warfarin

**DOI:** 10.7759/cureus.8114

**Published:** 2020-05-14

**Authors:** Ladan Panahi, Javier A Saenz II, Nam Nguyen, George Udeani, Salim Surani

**Affiliations:** 1 Pharmacy, Texas A&M Rangel College of Pharmacy, Kingsville, USA; 2 Pharmacy, Texas A&M Health Science Center, Kingsville, USA; 3 Internal Medicine, Corpus Christi Medical Center, Corpus Christi, USA; 4 Internal Medicine, University of North Texas, Dallas, USA

**Keywords:** tamoxifen, warfarin, inr, prothrombin, breast cancer, cancer anticoagulation, dvt prophylaxis

## Abstract

Tamoxifen causing an increase in the anticoagulation effect of warfarin is suggested to be clinically significant, but cases so far have been largely undocumented. Current recommendations advise clinicians to proceed with caution during concomitant therapy. In the presence of other medications known to interact with warfarin, such as antibiotics, proton pump inhibitors, amiodarone, and azole antifungals, international normalized ratio (INR) elevations can possibly be exacerbated even further. We hereby present a case report and a review of significant literature on the use of tamoxifen and warfarin concurrently.

## Introduction

Vitamin K antagonists have been well-studied for decades, with the most common drug utilized in this class being warfarin. Warfarin works by inhibiting vitamin K epoxide reductase (VKOR) enzyme. This enzyme functions to convert vitamin K from the oxidized, inactive form, to the reduced, active form [1]. Vitamin K-dependent clotting factors II, VII, IX, X in the body are synthesized via a mechanism requiring the reduced form of vitamin K. When VKOR is inhibited with warfarin use, this leads to a lack of vitamin K in its reduced form preventing clotting factors II, VII, IX, and X from forming. Due to the lack of these clotting factors being synthesized, the overall result observed is an anticoagulant effect. The prothrombin time (PT) is prolonged when the vitamin K-dependent factors II, VII, or X are decreased [1]. The interactions between the vitamin K antagonist, warfarin, and other medications are ever-present when initiating, maintaining, or managing multiple therapies, including tamoxifen. While tamoxifen therapy reduces incidences of breast cancer versus placebo, one of the significant adverse reactions seen in trials has been venous thromboembolism (VTE) with an incidence of up to 2% reported [1]. In the National Surgical Adjuvant Breast and Bowel Project P-1 (NSABP-1) trial, patients receiving tamoxifen without a past history of pulmonary emboli (PE) had a statistically significant increase in pulmonary emboli compared to placebo (RR-3.01, 95% CI: 1.15-9.27) [2]. Cancer places the patients at higher risk for deep vein thrombosis (DVT) as well and the addition of tamoxifen’s DVT risks further necessitates anticoagulant therapy in cancer patients. While anticoagulant therapy is imperative in cancer patients with recent venous thromboembolism (VTE), tamoxifen use with coumarin-type anticoagulants, such as warfarin has been documented to cause a significant increase in anticoagulant effect leading to a higher potential risk of bleeding [2]. The risk of bleeding is significant with warfarin use and can lead to several complications. During the period between 1993 and mid-Jul 2006, 9766 US bleeding cases attributed to warfarin were entered into the adverse reaction reporting system with 86% resulting in serious outcomes such as death, hospitalization, life-threatening, disability, congenital malformation, and 10% specifically had a fatal outcome [3]. Furthermore, a cohort study by Garcia-Rodriguez et al. showed patients undergoing warfarin therapy with an INR between 3.0 and 3.9 experienced a greatly increased risk of intracerebral hemorrhage (ICH) and subarachnoid hemorrhage (SAH) (odds ratio of 5.61, 95% CI 3.07-10.23; p-0.001) as compared to no therapy and patients with an INR greater than or equal to 3 had an odds ratio of 7.01 (95% CI 4.10-11.99) for ICH and an odds ratio of 2.64 (95% CI 0.95-7.35) for SAH [4]. Although literature is available regarding warfarin’s many drug interactions with specific drug classes, minimal data is available describing the interaction between warfarin with tamoxifen.

## Case presentation

A 79-year-old Caucasian woman presented to the emergency room after a motor vehicle collision. The patient suffered from multiple rib fractures, neck pain, and an open wound with a dislocated metatarsal joint. She had a blood pressure of 89/46 mmHg that improved with fluid resuscitation. She had a significant past medical history of metastatic breast cancer, nausea and vomiting, and deep venous thrombosis. Her significant past surgical history consisted of left breast lumpectomy, total abdominal hysterectomy, and ovarian cystectomy. For the past 15 months, the patient had been on tamoxifen 20 mg by mouth daily for metastatic breast cancer and on warfarin alternating between 4 mg to 6 mg by mouth daily for three years due to DVT. The patient’s INR report for the past year as an outpatient showed consistent therapeutic INR values at a goal of 2-3 until one month prior to admission where her INRs were subtherapeutic. The patient was placed on treatment doses of enoxaparin in the outpatient setting due to the subtherapeutic INR values and was restarted on the same enoxaparin regimen upon hospital admission. The patient’s home dose of tamoxifen and warfarin was restarted on day two of hospitalization. Her INR upon admission was 1.33 (Table 1). The patient underwent surgery for repair of her metatarsal joint on day one. On day four of hospitalization, her warfarin dose was reduced from 5 mg to 2 mg after an abrupt INR increase from 1.68 to 2.6 and her enoxaparin was placed on hold. On day five, her warfarin was held due to an increase in INR from 2.6 to 4.86. The patient’s warfarin was continued to be held thereafter but her INR continued to rise. One dose of vitamin-K 2.5 mg by mouth was administered on day eight of hospitalization when her INR peaked to a maximum of 8.28. The patient’s INR dropped to 1.33 the day after the oral vitamin K. Her enoxaparin was reinitiated at treatment doses (Table 1). Antibiotic coverage was expanded to cover hospital-acquired pneumonia. The patient improved and was discharged on day 12 to a skilled nursing facility on treatment doses of enoxaparin.

**Table 1:** Serial laboratory and medication data Hgb: hemoglobin; Hct: hematocrit; PT: prothrombin time; APAP: acetaminophen; SUBQ: subcutaneous; IV: intravenous

	Day 1 Admission	Day 2	Day 3	Day 4	Day 5	Day 6	Day 7	Day 8	Day 9	Day 10	Day 11	Day 12
Hgb (12 g/dL - 16 g/dL)	10.6	8.4	7.8	9.8	10.1	10.6	10.6	10.7	10.5	9.8	10.1	10.1
Hct (37% - 47%)	32.6	26.5	25.1	30.7	30.4	32.4	32.6	32.5	31.7	30.2	30.7	31
Platelets (150- 450 thou/cu mm)	305	233	226	172	192	236	291	317	322	305	364	378
PT (9.4 - 12.5 sec(s))	14.5	No lab	18.5	29.2	56.5	65.3	67.4	99	14.4	14.7	17.7	22.6
INR	1.33	No lab	1.68	2.6	4.86	5.58	5.75	8.2	1.33	1.35	1.61	2.03
In-Patient Medications Administered												
Warfarin	Held, surgery	6 mg	5 mg	2 mg	Held	Held	Held	Held	Held	Held	Held	Held
Enoxaparin	Held, surgery	70 mg SUBQ daily	70 mg SUBQ daily	Held	Held	Held			70 mg SUBQ daily	70 mg SUBQ daily	70 mg SUBQ daily	70 mg SUBQ daily
Phytonadione								2.5 mg po x 1				
Tamoxifen			20 mg po qday	20 mg po qday	20 mg po qday	20 mg po qday	20 mg po qday	20 mg po qday	20 mg po qday	20 mg po qday	20 mg po qday	20 mg po qday
Cefepime							1 gm IV x 1	1 gm IV q 12h	1 gm IV q 12h	1 gm IV q 12h	1 gm IV x 1	
Cefazolin	1 gm IV x 1 dose	1 gm IV q8h	1 gm IV q12h	1 gm IV q12h	1 gm IV q12h	1 gm IV q12h	1 gm IV x 1					
Clindamycin							600 mg IV x 2	600 mg IV q8h	600 mg IV q8h	600 mg IV q8h	600 mg IV x 1	
Vancomycin							1 gm IV x 1					
Metoclopramide										5 mg po x 2	5 mg po x 2, 5 mg IV x 1	5 mg IV x 4
Ondansetron			4mg IV x 1	4mg IV x 1	4mg IV x 1	4mg IV x 2	4 mg IV x 1	4 mg IV x 1	4 mg IV x 2	4 mg IV x 1		
Promethazine					6.25 mg IV x 1	6.25 mg IV x 1		6.25 mg IV x 1				
Hydrocodone/APAP			1 tab po	3 tab po	4 tab po	2 tab po		1 tab po	2 tabs po	3 tabs po	1 tab po	
Total APAP daily administered			325 mg	975 mg	1300 mg	650 mg		325 mg	650 mg	975 mg	325 mg	

## Discussion

Cancer patients have up to a four-fold higher risk of recurrent VTE and a two-fold higher risk of bleeding with many bleeding and thrombotic events occurring within the first four weeks of anticoagulant treatment [5]. Tamoxifen plays an important role in the current prevention and treatment of various types of breast cancer, including post-lumpectomy patients, yet the use of tamoxifen is associated with an increased risk of DVT [2]. A study investigating if tamoxifen leads to an increase in VTE showed a possible mechanism as a Factor V Leiden (FVL) mutation [6]. In that study, patients undergoing tamoxifen therapy for breast cancer had a VTE event and were five times more likely to have an FVL mutation [6]. Hypercoagulability due to cancer is a result of the direct activation of procoagulant pathways by cancer cells via the release of aberrant tissue factor (TF), TF-bearing microparticles, and cell surface proteases, as well as indirect activation by systemic effects of cancer on various cell types such as leukocytes endothelial cells and platelets [7]. Thus, the initiation of anticoagulant therapy is imperative in breast cancer patients on tamoxifen therapy. Warfarin has a narrow therapeutic index with specific goals for proper anticoagulation. An INR between 2 and 3 has shown the most acceptable reduction in the incidence of DVT with the least amount of bleeding events, while goals above 3 showed a higher incidence of bleeding without significant reductions in DVT [8]. Other factors, such as thrombocytopenia and organ or vascular invasion of tumors, in addition to the intensity of anticoagulation treatment, may be possible mechanisms to explain the increased bleeding observed in cancer patients receiving anticoagulation therapy [9].

In the maintenance of warfarin goals, understanding mechanisms of interactions can be helpful in managing interactions. Unfortunately, the exact mechanism of interaction between warfarin and tamoxifen remains unknown but the study by Boruban et al. suggests that tamoxifen inhibits CYP2C9 that is known to be the primary metabolizer of the S-isomer of warfarin [10]. CYP enzymes bind to foreign molecules, such as drugs, and form a reaction to increase water solubility for future excretion. These reactions can include N-dealkylation, O-dealkylation, aromatic hydroxylation, N-oxidation, S-oxidation, deamination, and dehalogenation [1]. The R-enantiomer is metabolized by CYP 2C19, 2C8, 2C18, 1A2, and 3A4. CYP2C9 metabolizes the S-enantiomer of warfarin by hydroxylation and reduction. The resulting dehydro-warfarin and warfarin alcohols have minimal anticoagulant activity and are excreted into the urine with a small amount excreted into the bile [1]. Some other proposed mechanisms for the drug interaction are protein binding, as both tamoxifen and warfarin are highly protein-bound as well as have tamoxifen-induced clotting factor effects [10].

As far as the guidelines for VTE management in cancer patients is concerned, CHEST guidelines suggest that patients at risk for recurrent VTE are those with reduced mobility, unprovoked VTE, and presence of active cancer [11]. For cancer patients with DVT, updated National Comprehensive Cancer Network (NCCN) guidelines recommend the initiation of direct oral anticoagulants (DOAC) or low molecular weight heparins (LMWH) as the preferred anticoagulant therapy for VTE in cancer patients as compared to vitamin K antagonists [9]. The guidelines recommend LMWH as the preferred treatment in patients with gastric or esophageal cancers due to an increased risk of hemorrhage with DOACs [9]. LMWH has shown to be more effective in vitamin k antagonists in reducing recurrent VTE in cancer patients [9,11]. Recent studies, such as the Anticoagulation Therapy in Selected Cancer Patients at Risk of Recurrence of Venous Thromboembolism (SELECT-D) trial and the Hokusai VTE Cancer trial demonstrated the non-inferiority of DOAC when compared to LMWH and reductions in major bleeding with the exception of patients with primary gastrointestinal and esophageal malignancies [7,12]. Patients with DVT and active cancer are recommended extended anticoagulant therapy for at least three months if the risk of bleeding is not high [9,11]. These patients should be reassessed annually or at more frequent periodic intervals [8]. The treatment and prevention of recurrent VTE is rarely discontinued in active cancer patients but when it is, there is an estimated annualized rate of 15% for VTE recurrence [5]. However, more studies are needed to assess the risks of discontinuing treatment and the level of risk variance based on the type of cancer, its progression, and type of treatment.

There have been few documentations of the drug-drug interaction between tamoxifen and warfarin. The case reports, retrospective reviews, and retrospective studies reported in the literature are summarized in Table 2.

**Table 2:** Literature Summary a. PT units reported in seconds; control unit reported in seconds INR: international normalized ratio; PT: prothrombin time; --- Data not reported

Reference	­­	Pts. N	Total weekly warfarin dose < 25 mg N (Dose)	Total weekly warfarin dose > 25 mg N (Dose)	Baseline PT ^a^ (control)	Tamoxifen dose	Concomitant use < 30 days N	Concomitant use > 30 days N (Days)	Maximum PT ^a^ (control)	Maximum INR	Time frame maximum PT /INR observed	Signs/symptoms bleeding present N (%)
Lodwick [13] 1987	Case Report	1		1 (27-28.5 mg)	23 to 34 (12)	10 mg twice daily	---	1	206 (14)	---	6 weeks	1/1 (100%)
Tenni [14] 1989	Case Report	1	----	1 (35 mg)	19 (--)	40 mg daily	1	---	38 (---)		1 day	0/1 (0%)
Tenni [14] 1989	Chart Review	5	3 (6 mg)	2 (25 mg)	---	---	5	---	49 to 50 (--)	---	---	2/5 (40%)
Ritchie [15] 1989	Chart Review	22	---	---	---	---	---	---	---	---	---	3/22 (14%)
Jönsson [16] (2007)	Chart Review	1	---	---	---	---	---	---	---	3.7	---	1/1 (100%)
Mishra [17] (2007)	Case Report	1	---	1 (35 mg)	---	20 mg daily	1	---	---	10.27	3 days	1/1 (100%)
Valachis [18] (2019)	Case-control study	1787	---	---	---	---	---	---	---	---	---	92/1787 (5%)

A comparison of detailed case reports to the case at hand shows some key similarities and differences. One observation that can be made is the impact of the concomitant use of warfarin and tamoxifen on PT and/or INR. Based on the given data, increases in PT and/or INR can be potentially observed with low or high weekly doses of warfarin and varied doses of tamoxifen ranging up to 20-fold increases in PT and eight to 10-fold increases in INR. Based on the reported data, an increase in PT and/or INR can be seen as early as one day after the concomitant use of both medications and can continue to increase up to weeks after concomitant use. One difference seen is the impact of interventions such as warfarin dose reductions on PT/INR values. Although Lodwick et al. and Tenni et al. reported success in reaching therapeutic PT/INR levels with dose reductions in warfarin with concomitant tamoxifen use, our case study, as well as that of Mishra et al., differed in the sense that the patient continued to have elevated PT/INR levels even after warfarin doses were reduced or held and not administered to the patient [13-14,17]. The majority of the cases and data reported observed differences in PT/INR and/or signs and symptoms of bleeding within 30 days of concomitant use of both tamoxifen and warfarin [13-14,17]. Another point to discuss is the variability of bleeding observed in the reported studies [13-18]. In the studies that reported bleeding and warfarin dosing, the majority of bleeding occurred when patients were given weekly warfarin doses greater than 25 mg while on tamoxifen therapy. No correlations in tamoxifen dose and bleeding occurrence can be made with the data reported from the studies listed in Table 2. The bleeding observed ranged from hematemesis, hematuria, subconjunctival hemorrhage, thigh hematoma, subdural hematoma, and cerebral hemorrhage. No correlations can be made in terms of the increase in PT/INR seen in these studies and the severity of bleeding. More studies and data would be needed to be able to determine if a correlation does exist. The cases uniformly did not discuss a third offending factor, overall hepatic or renal function, or significant past medical history pertinent to coagulability and bleeding.

Antibiotics may have potential effects on warfarin anticoagulation. This patient received cefazolin upon admission for an open wound secondary to a motor vehicle accident. Cephalosporins demonstrate a possible increase in INR [1,19]. It may be possible that these antibiotics interactions with warfarin, coupled with the interaction of tamoxifen, may have led to elevations in INR. The interaction between cefazolin and warfarin is possibly a contributor to the elevations observed in this case. A study by Baillargeon et al. showed cephalosporins demonstrated an increased incidence of bleeding (OR 2.45; 95% CI, 1.52-3.95) when used concomitantly with warfarin [19]. Literature examining the effects on INR by cefazolin specifically has not been produced.

The limitations of this study include the hepatic function of this patient after the motor vehicle accident may have not been thoroughly evaluated. A liver function test was done upon admission and showed an elevation in aspartate aminotransferase (AST) at 109 IUnits/L, with normal alanine transaminase (ALT) and low total bilirubin at 0.1 mg/dL. Blood urea nitrogen (BUN) was elevated at 28 mg/dL and a prolonged PT of 14.5 seconds was also observed. If further liver function tests (LFTs) had been done, possible elevations in AST/ALT may have been observed. Liver injury due to acetaminophen can be ruled out due to submaximal doses during the patient’s stay. The patient received an acetaminophen-containing product starting on day three and dosages remained under 2 grams through the patients' stay with a maximum dose of 1.3 grams on day five. Acetaminophen has shown increases in INR that is dose-dependent, as well as duration-dependent, with effects being seen after 10 to 14 days of use [1]. The patient received less than 10 days of acetaminophen during their hospital stay. Therefore, the acetaminophen used concomitantly with warfarin may have played a potentially minor role in the INR elevation observed with this patient.

## Conclusions

The use of tamoxifen with warfarin resulted in this patient’s elevation of INR witnessed during hospitalization. Further studies on increased anticoagulation by warfarin due to interaction with tamoxifen may be warranted to establish stronger recommendations regarding concomitant use.
